# Impairment of Autophagy Causes Superoxide Formation and Caspase Activation in 661 W Cells, a Cell Line for Cone Photoreceptors, under Hyperglycemic Conditions

**DOI:** 10.3390/ijms21124240

**Published:** 2020-06-14

**Authors:** Koichiro Taki, Taeko Horie, Teruyo Kida, Masashi Mimura, Tsunehiko Ikeda, Hidehiro Oku

**Affiliations:** Department of Ophthalmology, Osaka Medical College, Osaka 569-8686, Japan; k_taki0427@yahoo.co.jp (K.T.); opt168@osaka-med.ac.jp (T.H.); opt038@osaka-med.ac.jp (T.K.); opt119@osaka-med.ac.jp (M.M.); tikeda@osaka-med.ac.jp (T.I.)

**Keywords:** diabetic retinopathy, autophagy, mitophagy, 661 W cell, 3 methyl adenine, rapamycin

## Abstract

Microvascular changes are the earliest adverse events in diabetic retinopathy, but recent studies have shown that oxidative stress induced by photoreceptors is associated with the development of the retinopathy. The purpose of this study was to determine the roles played by superoxides formed by photoreceptors under hyperglycemic conditions on autophagy. To accomplish this, we cultured 661 W cells, a transformed murine cone cell line, with 5.5 or 25 mM glucose in the presence or absence of 3 methyl adenine (3MA) or rapamycin. The superoxides were determined by flow cytometry using hydroethidine as a fluorescence probe. The autophagy activity was determined by changes in the expression of LC3B2 and P62 by immunoblotting. The degree of mitophagy was determined by the accumulation of mitochondria and lysosomes. Apoptotic changes of 661 W cells were determined by the caspase 3/7 activities. Our results showed higher levels of P62 and superoxides in cells cultured in 25 mM glucose than in 5.5 mM glucose. Addition of 3MA caused a significant increase of P62, superoxides, and caspase 3/7 activities in the 661 W cells cultured in high glucose but not in low glucose. These findings suggest that autophagy is important for the functioning and survival of 661 W cells under hyperglycemic conditions.

## 1. Introduction 

Diabetic retinopathy is one of the leading causes of the blindness worldwide [1]. Microvascular damage of the retinal vessels occurs in the early phases of diabetic retinopathy which includes a loss of the pericytes [2], alterations in the retinal hemodynamics [3], and a breakdown of the blood retinal barrier [4]. These events impair the functioning of autoregulatory mechanisms in the retinal circulation, and the blood supply cannot adapt to the retinal demand causing retinal ischemia [5].

Diabetic retinopathy seldom occurs in cases of retinitis pigmentosa [6], and diabetes-related retinal changes are absent in diabetic Rho–/– mice [7]. These findings suggest that the photoreceptors play a critical role in the development of diabetic retinopathy [8]. On the other hand, it has been shown that the reactive oxygen species (ROS) are involved in the pathogenesis of diabetic retinopathy because the presence of antioxidant substances depresses the development of diabetic retinopathy [9]. Because the demand for oxygen is highest for the photoreceptors, the retinal ROS are chiefly derived from the mitochondria in the photoreceptors [10].

Autophagy is a catabolic system that recycles metabolic products, and it acquires an energy source during starvation. However, defective organelles including mitochondria can be eliminated by autophagic degradation which is specified as mitophagy [11]. Defective organelles including mitochondria are ubiquitinated by binding to p62/sequestrome 1 (SQSTM1) that are linked to LC3-forming autophagosomes. Autophagosomes carrying ubiquitinated mitochondria are fused with the lysosomal membrane via the lysosome membrane-associated protein 2A (LAMP2A) and constitute autolysosomes. Defective mitophagy causes functional damages of the mitochondria including a reduction in the mitochondrial membrane potentials, impairment of oxidative phosphorylation, increased production of superoxides, and release of cytochrome C [12]. These events are closely associated with oxidative stress [13]. Although the role played by autophagy in the central nervous system is still not completely determined, autophagy is important for neuronal cells lacking the ability of cell division because harmful substances can also be eliminated by cell division [14]. Autophagy is reportedly compromised in diabetic conditions [15], leading to an increase in the superoxide formation [16,17].

The purpose of this study was to determine whether impaired mitophagy under high glucose conditions will increase the formation of superoxides from the accumulation of defective mitochondria in the photoreceptors. To accomplish this, we cultured 661 W cells, a transformed murine cone cell line, with 5.5 or 25 mM glucose concentrations in the presence or absence of 3 methyl adenine (3MA), an autophagy inhibitor. The 661 W cells were also cultured with rapamycin, an autophagy inducer. We then determined the changes in the superoxide levels in the 661 W cells under these different culture conditions. In addition, the activities of autophagy were determined by the changes of LC3B and P62 expressions as well as mitochondrial and lysosomal accumulation by cytochemistry. The apoptotic changes of 661 W cells were determined by the caspase 3/7 activities.

## 2. Results

### 2.1. Changes of p62 and LC3B

**Figure 1:**
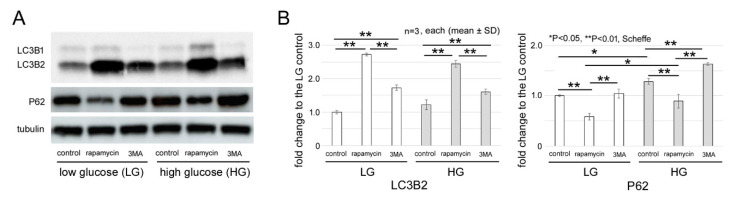
Western blots for LC3B2 and P62. (**A**) Representative protein bands of LC3B2 and P62 in extracts from 661 W cells cultured under different conditions. (**B**) Levels of LC3B2 and P62 are shown as fold changes (means ± SDs) relative to the low glucose (LG) control. Rapamycin increased the LC3B2 and decreased P62 levels significantly under both conditions indicating that autophagy was activated. P62 levels were higher under the high glucose (HG) control than those of the LG control, and addition of 3 methyl adenine (3MA), an inhibitor of autophagy, caused a further significant increase of P62 under HG conditions. (** *p* < 0.01, * *p* < 0.05, Scheffe; *n* = 3 each).

Representative immunoblots of LC3B and P62 are shown in Figure 1A. Their levels are shown by relative changes to ɑ-tubulin expression in Figure 1B. Cytosolic LC3-1 is converted to lipid-modified LC3-2, and then translocated into the autophagosome membranes [18]. Thus, the autophagic activities are more associated with the levels of LC3-2 than those of LC3-1 [19]. On the other hand, P62 is an adaptor protein of selective autophagy that increases when autophagy is impaired [20]. Thus, we determined the levels of LC3B2 and P62 under different culture conditions. The levels of both proteins are expressed as fold changes of the 661 W cells cultured in 5.5 mM glucose without addition of 3MA or rapamycin (low glucose (LG) control level).

The level of the LC3B2 of the high glucose (HG) control increased by 1.22 ± 0.06-fold from the LG control (*n* = 3, each) but this increase was not significant (*p* = 0.2). However, the P62 levels of the HG control were significantly (*p* = 0.02, Scheffe) increased to 1.3 ± 0.1-fold from the LG control.

The presence of 3MA increased the LC3B2 expression significantly by 1.7 ± 0.1-fold in 5.5 mM glucose (*p* < 0.01, Scheffe) and by 1.6 ± 0.1-fold in 25 mM glucose from the LG control levels. However, the P62 level was increased 1.3 ± 0.06-fold (*p* = 0.9) in 5.5 mM glucose and 1.6 ± 0.03-fold (*p* = 0.01, Scheffe) in 25 mM glucose. In spite of the similar LC3B2 levels in both conditions, 3MA caused a significant increase of P62 levels in the HG condition. This suggested that 3MA caused a greater impairment in autophagy under the HG condition.

Rapamycin similarly increased the LC3B2 levels significantly by 2.7 ± 0.05-fold in LG and 2.4 ± 0.1-fold in HG conditions (*p* < 0.01, Scheffe). In addition, rapamycin decreased the levels of P62 significantly under both conditions (*p* < 0.01, Scheffe). However, the decrease in the P62 levels was significantly greater in the LG condition (*p* = 0.01, Scheffe).

### 2.2. Changes of Mitophagy

**Figure 2:**
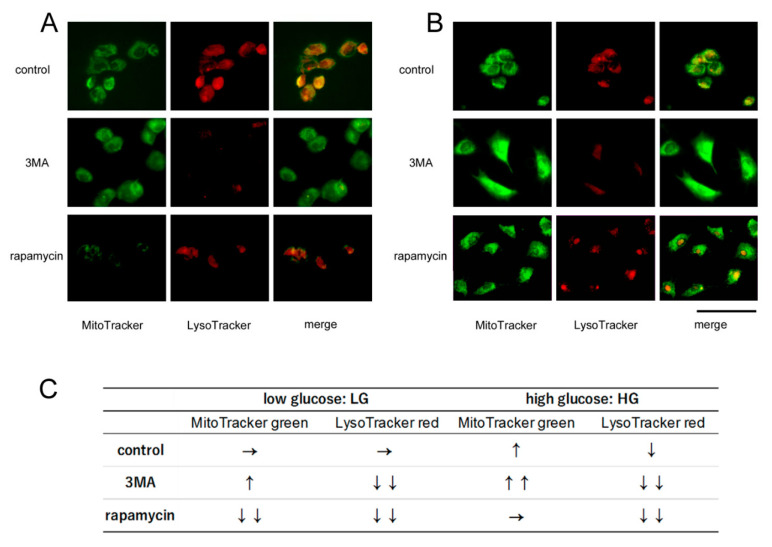
Cytological assessments of mitophagy. Representative photomicrographs using MitoTracker green^®^ and LysoTracker red^®^ dyes of 661 W cells cultured under low glucose (**A**) and high glucose (**B**) conditions. Mitochondria (green) and lysosomes (red) are made visible by these dyes respectively, and their colocalization is shown. Rapamycin, an inducer of autophagy, decreased the intensity of MitoTracker green fluorescence under LG condition indicating mitochondrial digestion (**A**, bottom), while this effect was almost absent under HG condition (**B**, bottom). The intensity of the MitoTracker green fluorescence appeared to be increased by an addition of 3MA, an inhibitor of autophagy under HG conditions (**B**, middle), while the increase in the intensity appeared to be modest under LG conditions (**A**, middle). These findings suggest mitochondrial digestion is enhanced by rapamycin in LG condition and is depressed by 3MA in HG conditions (bar = 100 µm). (**C**) Qualitative assessment of mitophagy using fluorescence probes under each condition. Upward and downward arrows indicate an increase or decrease in the intensities.

Representative fluorescence images of mitophagy are presented in Figure 2A,B and qualitative assessment is shown in Figure 2C. In the HG controls in the absence of rapamycin or 3MA (Figure 2B), the intensity of the green fluorescence of MitoTracker appears to be stronger while the red fluorescence of LysoTracker appears to be weaker compared to that of the LG control cells (Figure 2A).

Rapamycin decreased the green fluorescence intensity of MitoTracker in the cells cultured in LG, while 3MA seemed to intensify the green fluorescence of the MitoTracker and weakened the red fluorescence of LysoTracker in the cells cultured in LG condition (Figure 2A). Similar effects of 3MA were found under the HG condition. Thus, 3MA intensified the green fluorescence of the MitoTracker and reduced the red fluorescence of the LysoTracker. However, contrary to its effects in the LG condition, rapamycin did not decrease the green fluorescence intensities in the HG condition (Figure 2B).

### 2.3. Increase of Superoxide Formation

**Figure 3:**
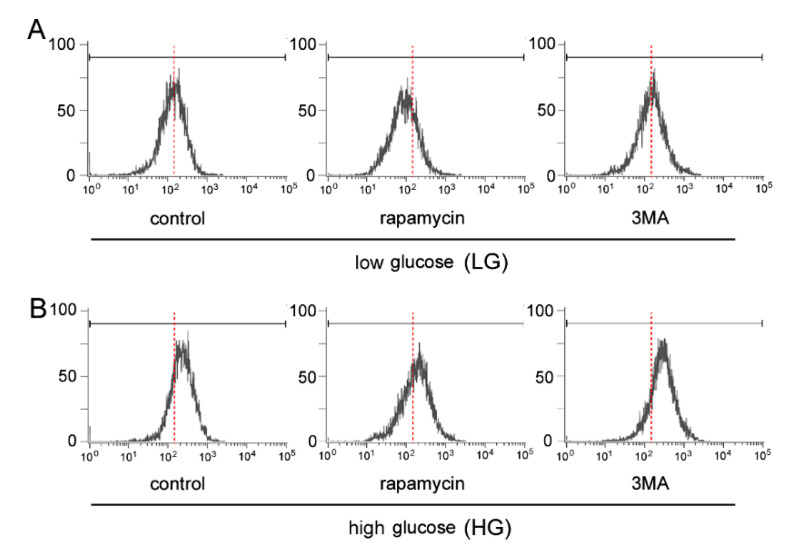
Intracellular superoxide formation by 661 W cells cultured under low glucose (**A**) and high glucose (**B**) conditions. Flow cytometric analyses of superoxide formation under each condition. Vertical axis represents the cell numbers and horizontal axis represents the ethidium fluorescein intensities. The red dotted lines indicate the mean fluorescein intensity of the LG control level. Fluorescence intensity was higher under HG control than LG control, indicative of increased superoxide formation under HG condition. Addition of 3MA, an inhibitor of autophagy, caused a further rightward shift under HG condition (**B**), while this effect was absent under LG condition (**A**). Rapamycin, an inducer of autophagy, slightly decreased the fluorescence intensity from the control under LG condition (**A**), while the effect was not seen under HG condition (**B**).

Intracellular superoxide formation in the 661 W cells cultured in different conditions was determined by the intensity of ethidium fluorescence by flow cytometry. Over 10,000 cells were analyzed, and their ethidium intensities are plotted on the horizontal axis and the number of cells on the vertical axis in Figure 3. Compared to the LG control, rapamycin caused a leftward shift in the LG condition. 3MA did not cause the rightward shift in the LG condition, but 3MA caused a further rightward shift in cells cultured under the HG condition.

**Figure 4:**
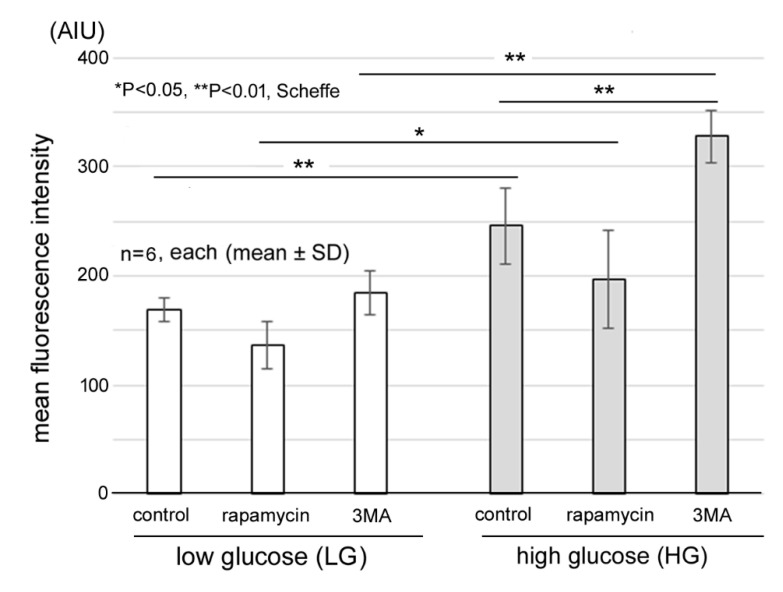
Quantitative analyses of intracellular superoxide levels. The intracellular superoxide levels are shown by fluorescein intensities in arbitrary intensity units (AIUs). Superoxide levels are significantly higher under HG control than LG control. Similarly, superoxide levels are significantly higher between other corresponding groups under HG condition than LG condition. Inhibition of autophagy by 3MA caused a further significant increase under HG condition while it did not increase the level under LG condition. Rapamycin slightly decreased the fluorescein intensities from the control in both conditions, but the effect was not significant. ** *p* < 0.01, * *p* < 0.05, Scheffe; *n* = 6 each.

The quantitative assessments from two independent experiments are shown in Figure 4. In each experiment, cells from three culture dishes under each condition were analyzed (each total *n* = 6). The mean fluorescence intensity of the LG control samples was 169.2 ± 10.4 arbitrary intensity units (AIUs). In the LG condition, the addition of 3MA or rapamycin did not change the mean fluorescence intensities significantly, and the levels were not significantly different in each condition.

On the other hand, the fluorescence intensity in the HG control was 245.8 ± 35.0 AIUs, which was significantly higher than that for the LG control (*p* = 0.01, Scheffe). The addition of 3MA caused a further significant increase (*p* = 0.003, Scheffe) from the HG control, and the level was 327.8 ± 23.6 AIUs. Rapamycin decreased the fluorescence intensity to 196.7 ± 45.0 AIUs, but this reduction was not significant (*p* = 0.2).

### 2.4. Activities of Caspase 3/7

**Figure 5:**
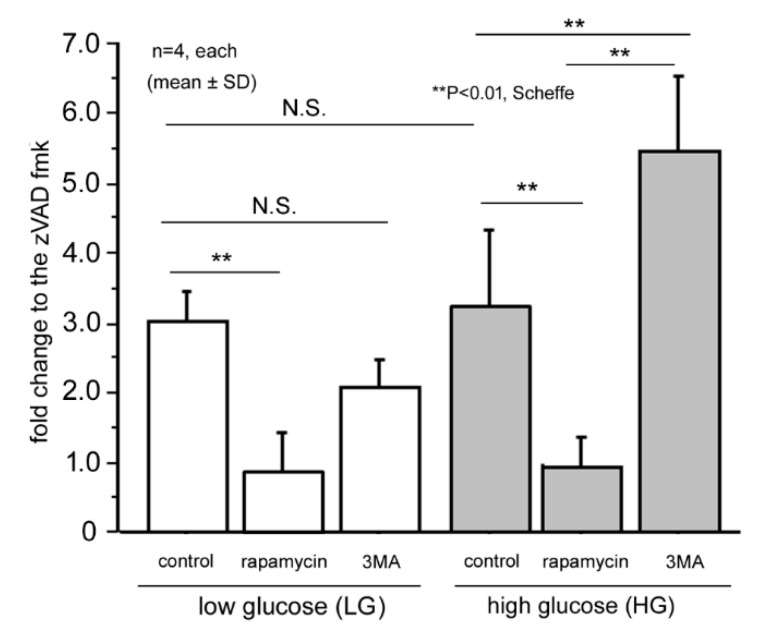
Caspase 3/7 activities in each condition. Data are expressed by the fold changes to the zVAD fmk control. The levels were similar between the LG and HG controls. Rapamycin decreased the activities to the levels of zVAD fmk control under both conditions. Inhibition of autophagy by 3MA increases the caspase activities under HG condition, while the levels were unchanged under LG condition. Thus, impairment of autophagy by 3MA increased apoptotic changes of 661 W cells under HG conditions. ** *p* < 0.01, Scheffe; *n* = 4 each.

The activities of caspase 3/7 were determined by ApoOne^®^ after 48 h of culture under different conditions. The results are expressed by the fold changes relative to the cells treated with Z-VAD-FMK, a pan-caspase inhibitor, in Figure 5.

In the physiological glucose concentration of 5.5 mM, the mean caspase 3/7 activity of the LG control was 3.0 ± 0.5-fold and the addition of rapamycin significantly decreased the level to 0.9 ± 0.3 (*p* = 0.002, Scheffe). However, 3MA did not increase the activities and the mean level was 2.1 ± 0.4-fold (*p* = 0.7, Scheffe). In the HG condition, the mean caspase 3/7 activity was 3.3 ± 1.1-fold, and the level was not significantly different from the LG control (*p* = 0.99, Scheffe). Rapamycin decreased the levels significantly to 0.8 ± 0.3-fold (*p* = 0.003), and 3MA increased the levels significantly to 5.5 ± 1.1-fold (*p* = 0.01, Scheffe). These results indicated that autophagy was more important for cellular survival under the HG condition.

## 3. Discussions

Our results showed the 661 W cells cultured in 25 mM glucose media, HG media, without addition of rapamycin or 3MA, had higher levels of P62 and superoxide formation compared to those cultured in 5.5 mM glucose (LG) media as control. The addition of 3MA caused a further significant increase of P62, superoxide formation, and caspase 3/7 activities in the 661 W cells cultured in HG, and these changes were not observed in those cultured in LG. Based on these observations, we suggest that autophagy is more important for 661 W cells to function and survive in HG condition than in LG condition.

We assessed the autophagic function by the changes of LC3B2 and P62 using immunoblotting. Rapamycin significantly increased the LC3B2 levels and decreased the P62 levels which indicated an activation of autophagy under both LG and HG conditions. However, the P62 levels were significantly higher under HG condition and the effects of rapamycin were higher under LG conditions. In addition, 3MA increased the LC3B2 levels under both conditions.

Class III PI3K signaling pathway is a positive regulator of autophagy [21], and 3MA, a class III PI3K inhibitor, is widely used as a pharmacological inhibitor of autophagy [22]. While 3MA inhibits the class I PI3K signaling pathway continuously, the inhibition of class III PI3K signaling pathway is transient [23]. Thus, the effects of 3MA on autophagosome formation appears to be complex, and 3MA may increase the LC3B2 level under some conditions [23]. On the other hand, the effects of 3MA on the P62 levels depended on the culture conditions, i.e., 3MA significantly increased the P62 level under HG conditions but it did not cause significant changes under LG conditions. Because autophagy is activated by starvation and is depressed by nutrition-rich conditions, the basic levels of the activities were different between LG and HG conditions which may account for the different actions of 3MA on P62 expression as determined by immunoblotting.

Digestion of one-half of the autophagosomes carrying mitochondria is completed within 7.5 min after activation of mitophagy [24]. Thus, we assessed the degree of mitophagy cytologically 30 min after the addition of 3MA or rapamycin. The two dyes, MitoTracker green and LysoTracker red, were well colocalized in the LG controls suggesting that these mitochondria were active during the process of mitophagy (Figure 2A, top). In the HG control, it appeared that higher MitoTracker green and lower LysoTracker red fluorescence were observed compared to the LG control (Figure 2B, top). Because blocking the autophagy depresses an increase of LysoTracker red fluorescence after the stimulation of autophagy [24], the increased LysoTracker red fluorescence may reflect the increase in the activities of autophagosome and autolysosome [24]. Considering the higher P62 levels, lower fluorescence intensities of LysoTracker red in the HG condition might suggest that the autophagic activities are depressed compared to the LG condition. In addition, higher intensities of MitoTracker green fluorescence in the HG control (Figure 2B, top) probably reflects a delay of degradation of the mitochondria.

It is possible that 3MA decreased the intensity of the LysoTracker red fluorescence under both HG and LG conditions by an inhibition of autophagy. However, the action of 3MA on the MitoTracker green fluorescence was different; 3MA further intensified the MitoTracker green in the cells cultured under HG condition (Figure 2B, middle), while the effects of 3MA seemed to be weaker under the LG condition (Figure 2A, middle). Thus, it is likely that more defective mitochondria would be accumulated under the HG condition after the addition of 3MA.

The effects of rapamycin on the MitoTracker green fluorescence were also different. Rapamycin almost completely eliminated the MitoTracker green fluorescence together with a reduction of the LysoTracker red fluorescence under the LG condition (Figure 2A, bottom) 30 min after the addition. These findings probably reflect the disappearance of autophagosomes and autolysosomes due to a complete autophagic digestion. In contrast, MitoTracker green fluorescence appeared unchanged from the control under HG condition (Figure 2B, bottom). We suggest that this may be due to a delay of autophagic digestion.

It has been shown that acute hypoglycemia causes apoptotic death of 661 W cells [25,26]. Under this condition, hypoglycemia increased the LC3-2 levels reflecting autophagy induction, but lysosomal degradation was impaired through a reduction of lysosomal-associated membrane protein 2A (LAMP2A) that resulted in a failure of the energy supply through autophagy [26]. Our results showed that 3MA caused apoptotic death of 661 W cells under hyperglycemic conditions as determined by caspase 3/7 activities, while hyperglycemia alone did not increase the caspase activities. These findings are in good agreement with a report that showed that HG condition alone does not affect the cellular viabilities of ARPE-19, while 3MA causes death of ARPE-19 cultured under HG conditions [8].

We showed that 3MA caused a significant increase in the superoxide levels of 661 W cells under HG conditions, while it did not increase the levels under LG conditions. The HG condition may cause more accumulation of misfolded or unfolded proteins because the nutrition supply exceeds the cellular demands. Certain parts of the increased defective proteins are degraded by the endoplasmic reticulum (ER) [27] which may cause ER stress. Thus, autophagy is more important to supplement the ER function. Hyperglycemia impairs the electron transport chain leading to a leakage of electrons that increases the level of ROS and causes mitochondrial dysfunction [28,29]. Damaged mitochondria cannot synthesize ATP but can produce ROS leading to more mitochondrial damage [29]. Thus, higher amounts of mitochondria would cause a higher burden of mitophagy under HG conditions [28]. Addition of 3MA impairs mitophagy which leads to an accumulation of damaged mitochondria and the release of superoxides [30]. These findings, including, ours support the idea that mitophagy is more important under HG than LG conditions.

Although the precise mechanisms have not been determined, there is a close link between mitochondria and lysosome. It has been shown that mitochondrial dysfunction impairs lysosomal function which then results in autophagic dysfunction [31]. Although rapamycin increased the levels of LC3B2 and decreased the levels of P62 levels under both LG and HG conditions, rapamycin increased the MitoTracker green fluorescence and decreased LysoTracker red fluorescence under HG conditions. We suggest these differences may reflect a delay of mitophagy as described earlier, and mitochondrial dysfunction may account for the inconsistency.

There are several limitations in this study. Our findings were obtained entirely from a transformed murine photoreceptor cell line, and a direct translation of these results to the pathophysiology of the photoreceptors should be made cautiously. Further in vivo study is necessary to extend our findings to photoreceptors. A second limitation is that we did not determine time course of mitophagy. It would also be important to determine mitochondrial function or its membrane potentials. In addition, rapamycin could not depress the increase of P62 and superoxide formation completely. However, it decreased the caspase 3/7 activities to the levels of the Z-VAD-FMK control. These differences may reflect its neuroprotective effects independent of the mTOR pathway [32]. These issues need to be investigated further.

In conclusion, our results indicate that autophagy is most likely more important for 661 W cells under hyperglycemic conditions, and inhibition of autophagy would increase the superoxide formation and apoptosis. 

## 4. Materials and Methods

### 4.1. Chemicals

Unless noted, chemicals were purchased from Sigma-Aldrich (St. Louis, MO, USA). Rapamycin, an inhibitor of mammalian target of rapamycin (mTOR), was purchased from Tokyo Chemical Industry (Tokyo, Japan). Dulbecco’s Modified Eagle Medium (DMEM) with low glucose (d-glucose 1000 mg/L) and high glucose (d-glucose 4500 mg/L) was purchased from Gibco (Carlsbad, CA, USA). The concentrations of glucose were equivalent to 5.5 and 25 mM, respectively.

### 4.2. 661.W Cell Line

The 661 W is a transformed cell line of cone photoreceptors derived from mouse that has been immortalized by simian virus (SV) 40 T antigen [33]. The 661 W cell line expresses various cone specific proteins [34].

Cultures of 661 W cells were maintained in DMEM supplemented with 10% fetal bovine serum (FBS), 100 U/mL penicillin, and 100 μg/mL streptomycin (Gibco) in a humidified atmosphere of 95% air and 5% CO_2_ at 37 °C. The cells were passaged by trypsinization every 2 days because their doubling rate was 1.1 days [34]. The 661W cells at passages 16 to 22 were used in this study.

For the experiments, the cells were plated in 10 cm culture dishes or in 24- or 96-well culture plates in DMEM with 5.5 or 25 mM glucose (Gibco) at a density of 1 × 10^4^ cells/mL. The concentration of FBS was reduced to 2.0%. The cells were cultured for 48 h and then 10 µM rapamycin or 10 mM of 3 methyl adenine (3MA) was added to modulate the activities of autophagy and further incubated for a designated time. Thus, experiments were conducted under 6 different conditions: cells without chemicals cultured in 5.5 mM (low glucose control: LG control) or 25 mM glucose (high glucose control: HG control), and those with addition of rapamycin or 3MA. Bovine serum (2.0%) was removed from the media and 0.1% bovine serum albumin (BSA) was added in all groups before the addition of rapamycin or 3MA.

### 4.3. Immunoblotting for Expression of p62 and LC3B

661 W cells were cultured in either LG or HG for 48 h, and then were further cultured with or without rapamycin or 3MA overnight. They were then lysed with a cell lysis buffer containing 1.0 % SDS, 20 mM EDTA, and 50 mM Tris-Hcl in 100 mM saline. The suspension was centrifuged, and the supernatant was used to determine the total protein concentration by the Bio-Rad protein assay (Bio-Rad, Hercules, CA, USA). Samples were added to a 10% or 18% SDS-polyacrylamide gel and were transblotted onto PVDF membranes. The membranes were blocked with 5% non-fat dry milk in PBS-T (pH 7.2, 0.1 % Tween 20) followed by overnight incubation at 4 °C with mouse monoclonal anti-LC3B (Medical & Biological Laboratories, Nagoya, Japan) and rabbit polyclonal anti-P62 (abcam, Cambridge, UK). In addition, α-tubulin (Merck Millipore, Darmstadt, Germany) was used as an internal control.

The protein bands were visualized by a reaction with horseradish peroxidase conjugated to appropriate secondary antibodies (Promega, Madison, WI, USA). The intensities of signals were increased with an ECL plus Western blotting detection system (GE Healthcare, Buckinghamshire, UK). The signals were detected and documented with the densitometry imaging system (Fusion System, M&S Instruments Inc., Osaka, Japan).

### 4.4. Estimation of Mitophagy

The degree of mitophagy can be estimated by the expression of mitochondria and their colocalization with autophagic machineries [24]. Because autophagosomes that bind to damaged mitochondria are degraded by lysosomes, we made the mitochondria visible by the MitoTracker green^®^ dye (Thermo Fisher, Waltham, MA, USA). We then determined whether they were colocalized with lysosomes to determine how HG conditions affected the mitophagy. Because mitochondrial degradation is known to process rapidly after autophagic stimulation, we determined the early changes of mitophagy after addition of an autophagy inducer or inhibitor [24]. For this, 661 W cells were cultured in 24-well culture plates for 48 h either in 5.5 or 25 mM glucose media. Then, the cells were incubated with MitoTracker green^®^ dye and LysoTracker red^®^ dye (Thermo Fisher. 100 nM each) for 30 min. After completion of the dye loading, the cells were incubated in the presence or absence of rapamycin (10 µM) or 3MA (10 mM) for another 30 min. Then, the changes in the degree of mitophagy were photographed with a fluorescence microscope (BZ X700; Keyence, Osaka, Japan).

### 4.5. Determination of Superoxide Formation

Superoxide formation within the 661 W cells was measured by specific fluorogenic probe of hydroethidine. Hydroethidine is oxidized by superoxide to a fluorescent product, ethidium, which is retained intracellularly which allows a semiquantitative estimation of the cellular superoxide production [35].

After incubation for 48 h in different conditions, rapamycin (10 µM) or 3MA (10 mM) were added into some culture dishes, and they were further cultured in the presence or absence of rapamycin or 3MA overnight. Cells were harvested by trypsinization and hydroethidine (1 µg/mL; 3.2 μM) was loaded for 30 min. After centrifugation and washing with PBS, the cells were resuspended in phenol red-free DMEM at a density of 2.0 × l0^5^ cells/mL. The intracellular level of superoxide was measured by flow cytometry (EC800 Analyzer; Sony Biotechnology, Inc., Tokyo, Japan) at 488-nm excitation and 590- to 610-nm emission wavelengths.

### 4.6. Determination of Caspase Activity

661W cells (5,000 cells/ 100 μL) were cultured in black, clear bottom 96-wells culture plate (Iwaki, Tokyo, Japan) under the different conditions mentioned. The caspase-3/7 activity was measured with the Apo-ONE^®^ Homogeneous Caspase-3/7 Assay kit (Promega, Madison, WI, USA) following the manufacturer’s protocol. This assay is based on fluorescent signals from Z-DEVD-R110 which is cleaved by caspase-3/7 to rhodamine 110.

Briefly, after 48 h of incubation in 5.5 or 25 mM glucose in 2.0% FBS, the serum was removed from the media and 661 W cells were further incubated overnight with or without the addition of rapamycin or 3MA. Then, Apo-One Homogeneous Caspase-3/7 reagent (100 μL) was added to each well at room temperature. In some wells, 100 µM of the pancaspase inhibitor zVAD FMK (addition of 2.0 μL from 100 mM stock solution), was added before the addition of the reagent, and they were designated as zVAD FMK control. The plates were shaken at 500 rpm for 30 s, and the caspase-3/7 activities were determined by the fluorescence intensity (excitation 480 nm, emission 520 nm) using the GloMax^®^ Discover System (Promega, Madison, WI, USA). The activities of caspase3/7 in each condition are presented as the fold changes compared to the zVAD FMK control.

### 4.7. Statistical Analyses

The data are shown by the means ± standard deviations (SDs). Statistical analysis was performed by one-way analysis of variance (ANOVA) followed by Scheffe post-hoc test for comparisons among groups. The level of *p* < 0.05 is considered significant.

## Abbreviations

Rho	Rhodopsin
ROS	Reactive oxygen species
LC3	MAP1 LC3: Microtubule-associated protein light chain 3
SQSTM1	Sequestrome 1
LAMP2A	Lysosome membrane-associated protein 2A
3MA	3 methyl adenine
LG	Low glucose
HG	High glucose
AIU	Arbitrary intensity units
Z-VAD-FMK	Benzyloxycarbonyl-Val-Ala-Asp (OMe) fluoromethylketone
PI3K	Phosphoinositide 3-kinase
ARPE-19	A human retinal pigment epithelial cell Line
ER	Endoplasmic reticulum
ATP	Adenosine triphosphate
mTOR	mammalian target of rapamycin
DMEM	Dulbecco’s Modified Eagle Medium
FBS	Fetal bovine serum
BSA	Bovine serum albumin
EDTA	Ethylenediaminetetraacetic acid
PVDF	Polyvinylidene difluoride
Z-DEVD-R110	(Z-Asp-Glu-Val-Asp)2-rhofamine 110
PBS	Phosphate-buffered saline
